# Effectiveness of step-down versus outpatient dialectical behaviour therapy for patients with severe levels of borderline personality disorder: a pragmatic randomized controlled trial

**DOI:** 10.1186/s40479-018-0089-5

**Published:** 2018-07-10

**Authors:** Roland Sinnaeve, Louisa M. C. van den Bosch, Leona Hakkaart-van Roijen, Kristof Vansteelandt

**Affiliations:** 1 GGZ Rivierduinen - Centre for Personality Disorders Jelgersma, Rhijngeesterstraatweg 13-C, 2342 AN Oegstgeest, The Netherlands; 2 Erasmus School of Health Policy and Management (ESPHM) and institute for Medical Technology Assessment (iMTA), Erasmus University Rotterdam, Postbus 1738, 3000 DR Rotterdam, The Netherlands; 3 Z.org KU Leuven, University of Leuven, Leuvensesteenweg 517, 3070 Kortenberg, Belgium; 4 Present address: Scelta, Deventerstraat 459, 7323 PT Apeldoorn, The Netherlands

**Keywords:** Borderline personality disorder, Dialectical behaviour therapy, Residential treatment, Suicide, Self-injurious behaviour, Randomized controlled trial, Cost effectiveness

## Abstract

**Background:**

Step-down dialectical behaviour therapy (DBT) is a treatment consisting of 3 months of residential DBT plus 6 months of outpatient DBT. The program was specifically developed for people suffering from severe borderline personality disorder (BPD). The present study examines the effectiveness and cost-effectiveness of step-down DBT compared to 12 months of regular, outpatient DBT.

**Methods:**

Eighty-four participants reporting high levels of BPD-symptoms (mean age 26 years, 95% female) were randomly assigned to step-down versus standard DBT. Measurements were conducted at baseline and after 3, 6, 9 and 12 months. The Lifetime Parasuicide Count and BPD Severity Index (BPDSI) were used to assess suicidal behaviour, non-suicidal self-injury (NSSI) and borderline severity. Costs per Quality Adjusted Life Year (QALY) were calculated using data from the EQ-5D-3L and the Treatment Inventory Cost in Psychiatric Patients (TIC-P).

**Results:**

In step-down DBT, 95% of patients started the program, compared to 45% of patients in outpatient DBT. The probability of suicidal behaviour did not change significantly over 12 months. The probability of NSSI decreased significantly in step-down DBT, but not in outpatient DBT. BPDSI decreased significantly in both groups, with the improvement leveling off at the end of treatment. While step-down DBT was more effective in increasing quality of life, it also cost significantly more. The extra costs per gained QALY exceeded the €80,000 threshold that is considered acceptable for severely ill patients in the Netherlands.

**Conclusions:**

A pragmatic randomized controlled trial in the Netherlands showed that 9 months of step-down DBT is an effective treatment for people suffering from severe levels of BPD. However, step-down DBT is not more effective than 12 months of outpatient DBT, nor is it more cost-effective. These findings should be considered tentative because of high noncompliance with the treatment assignment in outpatient DBT. Furthermore, the long-term effectiveness of step-down DBT, and moderators of treatment response, remain to be evaluated.

**Trial registration:**

www.clinicaltrials.govNCT01904227. Registered 22 July 2013 (retrospectively registered).

## Background

### Outpatient dialectical behaviour therapy: an efficacious treatment for BPD

Borderline Personality Disorder (BPD) is a severe and persistent mental disorder. Clinical hallmarks include emotional dysregulation, impulsivity, self-injury and chronic suicidal ideation [1]. The suicide rate is higher than that found in the general population [2]. A recent prospective study of the course and outcome of 290 inpatients diagnosed with BPD found a completed suicide rate of about 4% in the first 6 years of follow-up [3]. The diagnoses is associated with high burden of illness [4], poor social outcome [5, 6] and health provider stigma [7].

Dialectical Behaviour Therapy (DBT) was developed for chronically suicidal individuals diagnosed with BPD. The treatment strategies are rooted in Linehan’s emotion regulation (skills deficit) model, which states that dysfunctional behaviour in BPD can be explained as either consequences of pervasive emotion dysregulation or ways of coping with it [8, 9]. The first phase of DBT focusses on skills to stop the vicious circle of emotion dysregulation. Standard phase one DBT has five components. First, a weekly skills training to increase capabilities to be mindful, regulate emotions, tolerate frustration and be effective in interpersonal relationships. Groups complete the curriculum twice over the course of a year, creating a 1-year training program. Second, individual therapy to enhance motivation and to help apply DBT skills in daily life. A third component, telephone coaching, facilitates skills generalization between sessions. The fourth and fifth components include specific case management strategies and team meetings to help therapists stay motivated and competent [8, 9].

The efficacy of standard, outpatient DBT as a treatment for BPD has been demonstrated in randomized controlled trials (RCT) in academic settings [10–17]. Follow-up data indicated treatment gains were maintained 6 to 12 months after treatment [18–20]. It was also shown DBT remains effective when it is implemented in non-academic settings [21–24]. A meta-analysis about the effects of psychological treatments for BPD confirmed that DBT is helpful in reducing inappropriate anger and non-suicidal self-injury (NSSI) as well as in improving general functioning [25]. Research evaluating mechanisms of change found applying DBT skills in daily life mediates treatment effectiveness [26, 27].

### A recurring debate: inpatient and residential treatments for BPD

DBT was originally developed in an outpatient setting [8, 11]. Linehan argued that if the function of suicidal behaviour is the communication of distress, the desire for companionship or avoidance of some aversive reality in daily life, then being hospitalized may reinforce the suicidal behaviour and prevent patients from developing functional coping skills to address their problems [8]. At the same time, longitudinal studies demonstrate that patients diagnosed with BPD are more likely to be hospitalized than patients diagnosed with other mental disorders [28–30]. Bloom et al. argued some patients are not sufficiently engaged in outpatient treatment and exacerbations of symptoms can exceed what providers can manage in an outpatient setting [31], suggesting the importance of effective inpatient DBT. Bloom et al. synthesized findings from 11 pre-post studies on the efficacy of inpatient DBT. Most studies reported reductions in suicidal ideation, self-injurious behaviour and symptoms of depression and anxiety. However, caution is needed when interpreting these findings as none of the studies were RCT’s, few included a comparison group and most were plagued by sample size issues [31].

### Synthesis: residential treatment as a preparation for outpatient treatment?

Bloom et al. stated that examining the effectiveness of inpatient DBT as an intensive preparation for outpatient DBT is the next step in developing best-practice guidelines [31]. Along the same lines, we wondered if it was possible to improve the effectiveness of DBT by developing a step-down DBT-program using 3 months of residential DBT as an intensive orientation to 6 months of outpatient DBT [32]. We use the term ‘residential’ instead of ‘inpatient’ to clarify that the residence was a home-like environment where patients only stayed on weekdays [33]. Support staff were only present during office hours. The residential setting allowed us to adapt standard DBT protocol: DBT skills were trained in 3 months instead of 6 months, patients were reminded about their skills every weekday and extra program parts, aimed at practicing and generalizing skills, were added. We hypothesized that for individuals suffering from high levels of BPD-symptoms this 9-month, step-down DBT program would lead to a significantly larger decrease in suicidal behaviour, NSSI and total level of borderline symptomatology than 12 months of standard DBT. We also expected that step-down DBT would lead to fewer drop-outs and would be more cost-effective when estimated over a 12-month period [32]. To our knowledge, this is the first time that these hypotheses were evaluated in a randomized controlled trial.

## Methods

We conducted an RCT with a two (group) by five (time) repeated measures parallel design, without blinding. The sequence of randomization was concealed until interventions were assigned. The protocol was in accordance to the principles outlined in the Declaration of Helsinki, approved by the Institutional Review Board and registered in www.clinicaltrials.gov [32]. There are three differences between the study protocol in Trials and this report. First, the name of the residential program was changed from ‘inpatient DBT’ to ‘residential DBT’. Second, our study ended prematurely due to an unexpected close-down of the Centre for Personality Disorders Jelgersma (CPJ). Third, because of unforeseen waiting list issues, participants who were randomized to outpatient DBT had to wait longer before they met their therapist.

### Sample

Participants gave written informed consent. They had to meet the DSM-IV TR criteria for BPD (identical to the criteria in DSM-5), be 18–45 years of age, score higher than 24 on the Borderline Severity Index-IV (BPDSI-IV) and report at least one episode of self-injurious behaviour within the month before the intake. If there was no episode of self-injurious behaviour 1 month before the intake, then a BDSI-score of at least 30 was required to be eligible for the study. Exclusion criteria were limited to having a diagnosis of a chronic psychotic disorder, bipolar I disorder, intellectual disability, substance dependence requiring detoxification, involuntary psychiatric treatment, insufficient command of Dutch or living outside of travelling distance from the treatment center.

### Therapists and trainers

All therapists and trainers were psychologists, psychiatrists, nurses or social workers working at GGZ Rivierduinen (*n* = 30). DBT team members completed at minimum a 3-day training in DBT and received supervision from the senior researcher. Adherence was assessed with the 5-point DBT Expert Rating Scale (Linehan, Lockard, Wagner & Tutek: DBT Expert Rating Scale, unpublished). Treatment integrity greater than or equal to four was considered adherent. Fifteen percent of the sessions were assessed. Scores ranged between 3.6 and 4.1, with an average of 3.9. Both step-down DBT and outpatient DBT contained the five components of the treatment protocol [8, 11, 12]. The DBT-skills were taught according to the first version of the manual [8, 12]. The only adaption was that telephone consultation outside of the office hours was within the limitations set by the therapist.

### Treatments

The experimental treatment, step-down DBT, consisted of 3 months of residential DBT plus 6 months of outpatient DBT [32]. In residential DBT, support staff were present during office hours to help the patients apply DBT skills. Program parts were added, including: daily mindfulness classes, daily meetings about living together as a group, weekly drama therapy, weekly group sessions on validation skills and chain analyses, and fortnightly network training sessions together with family and friends. Limiting residential DBT to 3 months had several advantages, including enabling us to limit costs, make clear to participants that the goal was preparing for outpatient DBT and to compare our results with ‘modal inpatient DBT’ [31, 34, 35]. Controls received 12 months of standard, outpatient DBT, organized in three community mental health settings of GGZ Rivierduinen [32].

### Measurements

#### Intake interview

Participants were screened with the Vragenlijst Kenmerken Persoonlijkheid [36]. Presence of Axis 1 and Axis 2 disorders was assessed with the mini-International Neuropsychiatric Interview [37] and the Structured Clinical Interview for DSM Disorders [38]. These are DSM IV-TR diagnoses. Validated, semi-structured interviews for DSM 5 diagnoses were not available in Dutch. A Dutch translation of the Lifetime Parasuicide Count (LPC) was used to obtain detailed information about the nature, frequency and function of self-injurious behaviour (Comtois & Linehan: Lifetime Parasuicide Count: description and psychometrics, unpublished; van den Bosch: Vragenlijst Parasuicidaal gedrag, unpublished). The LPC makes a distinction between self-injurious behaviour with suicidal intentions (LPC Sui), without suicidal intentions (LPC NSSI) or ambivalent suicidal intentions (LPC Amb). Frequency of borderline symptoms in the previous 3-month period was assessed with the BPDSI-IV [39].

#### Repeated assessments

After randomization, assessments took place at baseline and after 3, 6, 9 and 12 months. The LPC and BPDSI were used to collect data on suicidal behaviour, NSSI and borderline severity in the past 3 months. Quality of life was assessed with the EQ-5D 3 level version (EQ-5D-3L). The health descriptions of this measure can be linked directly to empirical valuations of the general public, which allows utilities to be computed [40]. The Dutch tariff was used to calculate preferences for EQ-5D health states [41]. Direct medical costs and productivity costs were measured with the Treatment Inventory Cost in Psychiatric Patients [TiC-P] [42]. Unit costs were valued according to prices reported in the Dutch manual for cost research [43].

### Randomization

A computer program, developed by the Amsterdam Medical Centre, generated the sequence. To increase the likelihood of comparable treatment groups, a minimization method was used. Minimization variables were BPDSI score ≥ 40, total lifetime LPC score ≥ 14 and age.

### Statistical analyses

Comparisons of key demographic and clinical characteristics of the analyzed sample were performed with t-tests for normally distributed variables and with Wilcoxon two sample tests for variables that were not normally distributed. To examine the association between categorical variables, we used Chi-square tests and Fisher’s exact tests. In the repeated measurements analyses data from the LPC, subscales were dichotomized because they were right-skewed with excess zeros. A generalized linear mixed model (GLMM) with random intercepts with logistic link function was estimated for dichotomous outcome variables [44]. This allowed us to examine whether the probability (yes or no) of self-destructive behaviour changed over time. A linear mixed model (LMM) with random intercepts and slopes was estimated for the BPDSI total score. In these models, condition, time, and time x condition were included as fixed effects. Time was expressed as number of months passed since baseline. Model selection and inference were based on Likelihood Ratio and Wald tests [45]. The Kaplan-Meier statistic was used to examine whether the time to dropout was longer for patients in step-down DBT compared to outpatient DBT. Non-starters were excluded from this analysis.

The cost-effectiveness of step-down DBT was assessed by estimating an incremental cost-effectiveness ratio (ICER). In this case, the ICER was the difference in costs of both interventions divided by the difference in quality adjusted life years gained (QALY’s). QALY’s were estimated using the EQ-5D-3L scores. Details of procedures to calculate the direct medical costs, productivity costs (e.g. absenteeism and presenteeism) and the ICER can be found in the guidelines of costing studies [43]. Subsequently, we assessed the probability that step-down DBT is more effective than outpatient DBT by comparing the costs per QALY to the costs that are considered acceptable for severely ill patients in the Netherlands (i.e. € 80,000) [46]. Non-parametric tests were conducted since the data were not normally distributed. A bootstrap simulation was run for 5000 iterations to estimate 95% confidence intervals (CI) for a range of probable values for total costs, effects and ICERs.

## Results

### Participant flow

**Fig. 1:**
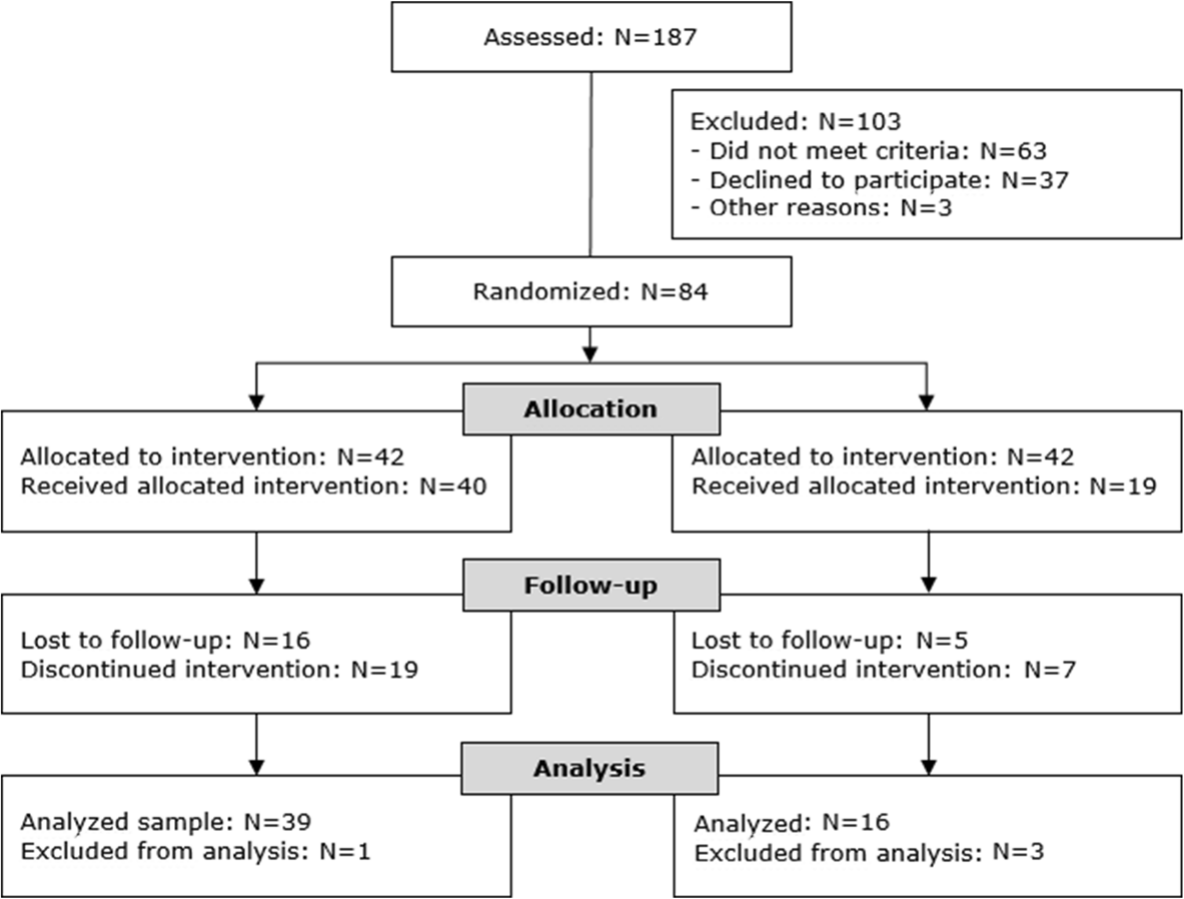
CONSORT flowchart pragmatic RCT step-down versus outpatient DBT. ‘Received the allocated intervention’ = Number of participants that attended at least one skills training or at least one individual therapy session after they signed a therapist-client agreement (referred to as ‘starters’ in the text). ‘Lost to follow up’= Number of participants that received the allocated intervention but did not complete a 12 month assessment. ‘Discontinued intervention’ = Number of participants that received the allocated intervention but dropped out before it was completely finished. In DBT, ‘dropout’ means that a participant missed four individual therapy or four weekly skills training session in a row. ‘Analyzed’ = Number of participants whose data were used to estimate statistical models for primary outcome variables

A total of 187 participants were assessed for eligibility from February 2012 to January 2014 (Fig. 1). Sixty-three participants did not meet the inclusion criteria. After randomization, two out of 42 participants in step-down DBT did not start the allocated treatment and one participant did not provide valid baseline data. Consequently, 39 participants were included in the primary analyses. In outpatient DBT, 23 out of 42 participants did not start the allocated treatment. This could be partially due to the fact that waiting times appeared to be longer in outpatient DBT. One participant died by suicide before he received outpatient DBT. Three out of 19 participants that started outpatient DBT did not provide valid baseline data.

### Characteristics analysed sample

The sample was 95% female. Fourteen participants were working (26%). An equal number was enrolled in college. Nineteen participants (35%) were considered unfit for work. The majority (*N* = 47, 85.5%) was divorced or single. Almost one third of the sample reported a history of sexual abuse (*N* = 16, 29%) and more than half experienced physical abuse (*N* = 30, 55%). One out of three participants suffered from posttraumatic stress disorder (*N* = 17, 31%), half were diagnosed with major depression (*N* = 28, 51%), and one out of three participants fulfilled criteria of substance dependence (*N* = 17, 31%). The average EQ-5D-3L score at baseline was .47 [standard deviation (SD) = .29], confirming an overall low quality of life. The total direct medical costs in the year before the study were high. The main cost drivers were admissions to psychiatric hospitals [€16,248 (SD = €32,838)] and psychotherapy [€5274 (SD = €7662)]. Other characteristics can be found in Table 1. There were no significant between-group differences in key demographic or clinical variables.

**Table 1:** Comparisons of key demographic and clinical characteristics in step-down DBT and outpatient DBT

	Step-down DBT^a^		Outpatient DBT^b^		Test Statistic	df	*p*
	M	SE	M	SE			
Age first mental health problems	11.13	.79	11.88	.67	*t* = −.72	49	.47
Age first contact mental health care	14.28	1.15	15.44	1.20	*t* = −.59	53	.56
Number of BPD criteria	7.10	.20	7.56	.30	*t* = −1.25	53	.22
Severity of BPD 3 m before intake	37.17	1.27	34.75	1.29	*t* = 1.12	53	.27
Age at time of intake	26.15	6.18^d^	25.63	7.45^d^	Z = −.71^e^	n.a.	.48
Suicide attempts lifetime	4.72	6.79^d^	25.84	52.90^d^	Z = −.64^e^	n.a.	.52
Ambivalent SI lifetime (*N* = 54)	12.50^c^	28.76^d^	33.88	77.59^d^	Z = .41^e^	n.a.	.68
NSSI lifetime (*N* = 44)	525.48^c^	876.74^d^	938.07^c^	1644.26^d^	Z = −.23^e^	n.a.	.82

*n.a.* not applicable; Comparisons performed with t-tests for normally distributed variables and with Wilcoxon two sample tests for variables that were not normally distributed. ^a^ *N* = 39; ^b^ *N* = 16; ^c^ Patients that persisted that they could not recall lifetime occurrence of self-injurious behaviours, because it was “too high to estimate” were removed from calculations; ^d^ Standard deviation (*SD*) was reported instead of standard error (*SE*); ^e^ Wilcoxon two sample tests

### Outcomes

#### Suicidal behaviour and NSSI

No completed suicides were recorded after participants started DBT. The probability of self-injurious behaviour with suicidal intention (LPC Sui), F(2, 156) = 2.90, *p* = .06, and with ambivalent suicidal intention (LPC Amb), F(2,156) = 2.63, *p* = .08, did not change significantly over 12 months. We found that the probability of self-injurious behaviour with suicidal intention, odds ratio (OR) = .33, 95% CI [.17–.63], F(1,32) = 12.28, *p* = .001, and with ambivalent suicidal intention, OR = .55, 95% CI [.38–.81], F(1, 32) = 10.00, *p* = .003, decreased during the 3 months of residential treatment in the step-down DBT group. This means that the hypothesis that a residential setting reinforces suicidal behaviour was rejected.

There were significant changes in the probability of NSSI (LPC NSSI) during treatment, F(2, 156) = 4.27, *p* = .02. More specifically, the probability of NSSI decreased significantly over 12 months in step-down DBT, OR = .90, 95% CI [.82–.98], t (156) = − 2.45, *p* = .02, but not in outpatient DBT, OR = .90, 95% CI [.79–1.03], t(156) = − 1.60, *p* = .11. Note that the difference between both groups is small. The fact that the OR is not significant in the outpatient DBT may be due to its smaller sample size. The estimated probabilities of self-injurious episodes during step-down DBT and outpatient DBT can be found in Table 2. The mean frequency of self-injurious episodes can be found in Table 3.

**Table 2:** Probabilities and 95% confidence intervals of self-injurious episodes based on a generalized linear mixed model

	Step-down DBT^a^			Outpatient DBT^b^		
	LPC Sui	LPC Amb	LPC NSSI	LPC Sui	LPC Amb	LPC NSSI
Baseline	.18	.26	.80	.29	.09	.80
	[.09–.33]	[.15–.42]	[.65–.89]	[.11–.58]	[.02–.29]	[.56–.93]
0–3 m	.17	.20	.74	.17	.11	.75
	[.09–.27]	[.12–.31]	[.61–.84]	[.07–.36]	[.04–.28]	[.54–.88]
3–6 m	.15	.15	.67	.09	.14	.69
	[.09–.25]	[.09–.25]	[.54–.78]	[.03–.24]	[.06–.29]	[.49–.83]
6–9 m	.13	.11	.60	.05	.17	.62
	[.07–.25]	[.05–.22]	[.44–.74]	[.01–.19]	[.07–.35]	[.40–.80]
9–12 m	.12	.08	.52	.02	.21	.54
	[.05–.27]	[.03–.21]	[.33–.71]	[.003–.16]	[.07–.47]	[.28–.78]

Timeframe = 3 months before measurement; Note: LPC Sui, LPC Amb, LPC NSSI = self-injury with respectively suicidal, ambivalent, non-suicidal intentions according to Lifetime Parasuicide Count; ^a^ Observations: *N*(Baseline) = 39, *N*(0–3 m) = 33, *N*(3–6 m) = 25, *N*(6–9 m) = 22, *N*(9–12 m) = 24; ^b^ Observations: *N*(Baseline) = 16, *N*(0–3 m) = 15, *N*(3–6 m) = 15, *N*(6–9 m) = 14, *N*(9–12 m) = 14

**Table 3:** Mean frequency and standard deviation of self-injurious episodes

	Step-down DBT^a^						Outpatient DBT^b^					
	LPC Sui		LPC Amb		LPC NSSI		LPC Sui		LPC Amb		LPC NSSI	
	M	SD	M	SD	M	SD	M	SD	M	SD	M	SD
Baseline	1.0	2.4	1.9	4.5	36.1	54.5	1.2	2.7	1.9	4.0	41.7	23.2
0–3 m	.0	.2	3.7	13.5	13.6	34.5	.1	.3	1.0	3.9	23.2	53.1
3–6 m	1.2	4.2	1.4	6.0	23.3	52.6	.1	.3	.5	1.1	15.7	30.8
6–9 m	.8	3.2	1.0	2.7	17.0	29.2	.2	.6	1.3	4.5	7.3	14.3
9–12 m	.2	.7	.0	.2	4.5	9.0	.5	1.9	.9	2.1	8.6	14.0

LPC Sui, LPC Amb, LPC NSSI = self-injury with respectively suicidal, ambivalent, non-suicidal intentions according to Lifetime Parasuicide Count; *M* mean, *SD* standard deviation; ^a^ Observations: *N*(Baseline) = 39, *N*(0–3 m) = 33, *N*(3–6 m) = 25, *N*(6–9 m) = 22, *N*(9–12 m) = 24; ^b^ Observations: *N*(Baseline) = 16, *N*(0–3 m) = 15, *N*(3–6 m) = 15, *N*(6–9 m) = 14, *N*(9–12 m) = 14

#### Drop-out

In step-down DBT, 53% of the participants who started DBT finished the entire 9-month program. Twelve months of outpatient DBT showed a retention rate of 63%. The results of the Kaplan Meier statistic indicated that there were no significant differences in the time to drop-out between conditions, Χ^2^(1) = .36, *p* = .55.

#### Severity of BPD

**Fig. 2:**
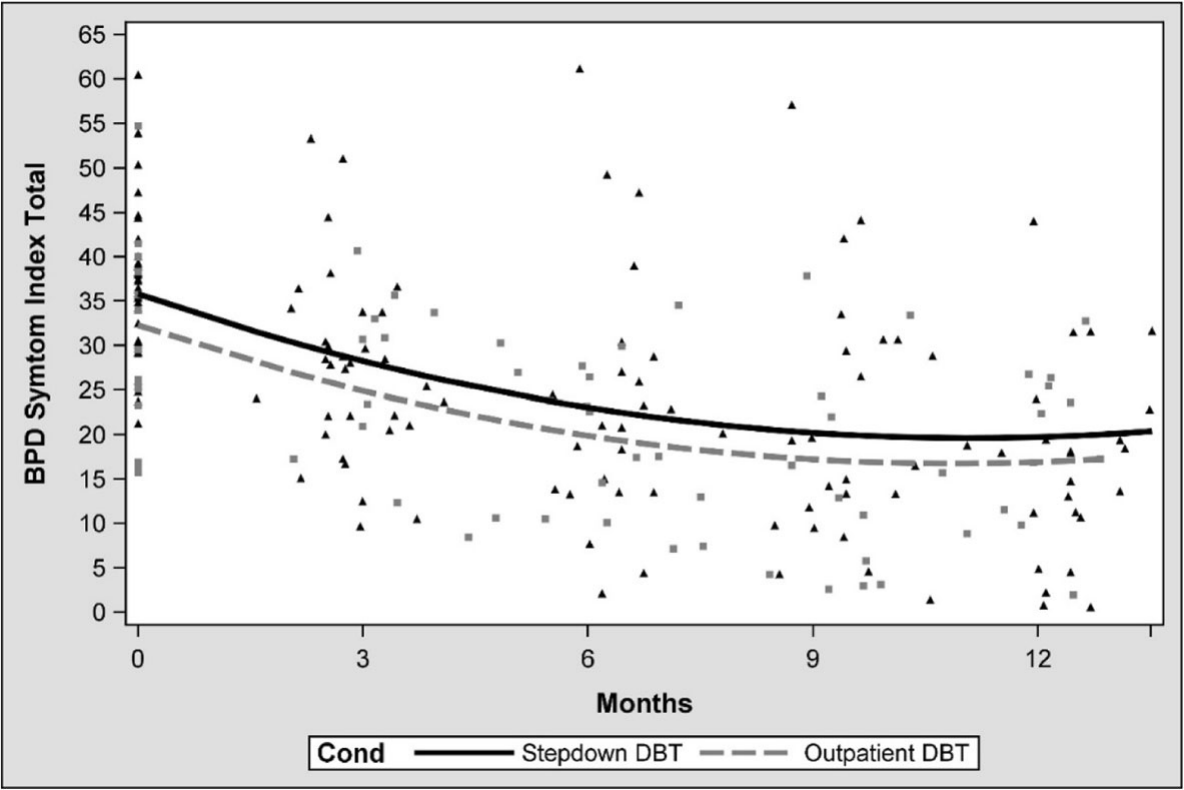
Estimated Borderline Personality Symptom Index score with time, condition and time x condition as predictors

The BPDSI total–score indicated that borderline symptomatology decreased significantly in both treatment groups, F(1, 109) = 33.63, *p* < .0001. The regression coefficients for months in step-down DBT and outpatient DBT were respectively − 2.87 (SE = .37), t(109) = − 7.86, *p* < .001, and − 2.82 (SE = .41), t(109) = − 6.82, *p* < .0001 (Table 4). This decrease levelled off near the end of treatment, F(1, 109) = 23.92, *p* < .0001. The regression coefficient of the quadratic effect of months was .1 (SE = .03) (Fig. 2).

**Table 4:** Borderline Personality Disorder Symptom Index: estimated means based on a linear mixed model

	Step-down DBT^a^			Outpatient DBT^b^		
	M	SE	95% CI	M	SE	95% CI
Baseline	35.80	1.38	33.07–38.52	32.27	2.14	28.02–36.52
0–3 m	28.32	1.38	25.58–31.06	24.97	2.10	20.81–29.13
3–6 m	23.14	1.68	19.82–26.47	19.97	2.41	15.19–24.75
6–9 m	20.27	1.98	16.33–24.20	17.27	2.87	11.58–22.95
9–12 m	19.69	2.44	14.85–24.52	16.86	3.51	9.91–23.82

*M* mean, *SE* standard error, *CI* confidence interval; ^a^ Observations: *N*(Baseline) = 39, *N*(0–3 m) = 33, *N*(3–6 m) = 25, *N*(6–9 m) = 22, *N*(9–12 m) = 24; ^b^ Observations: *N*(Baseline) = 16, *N*(0–3 m) = 15, *N*(3–6 m) = 15, *N*(6–9 m) = 14, *N*(9–12 m) = 14

#### Costs and cost-effectiveness

**Fig. 3:**
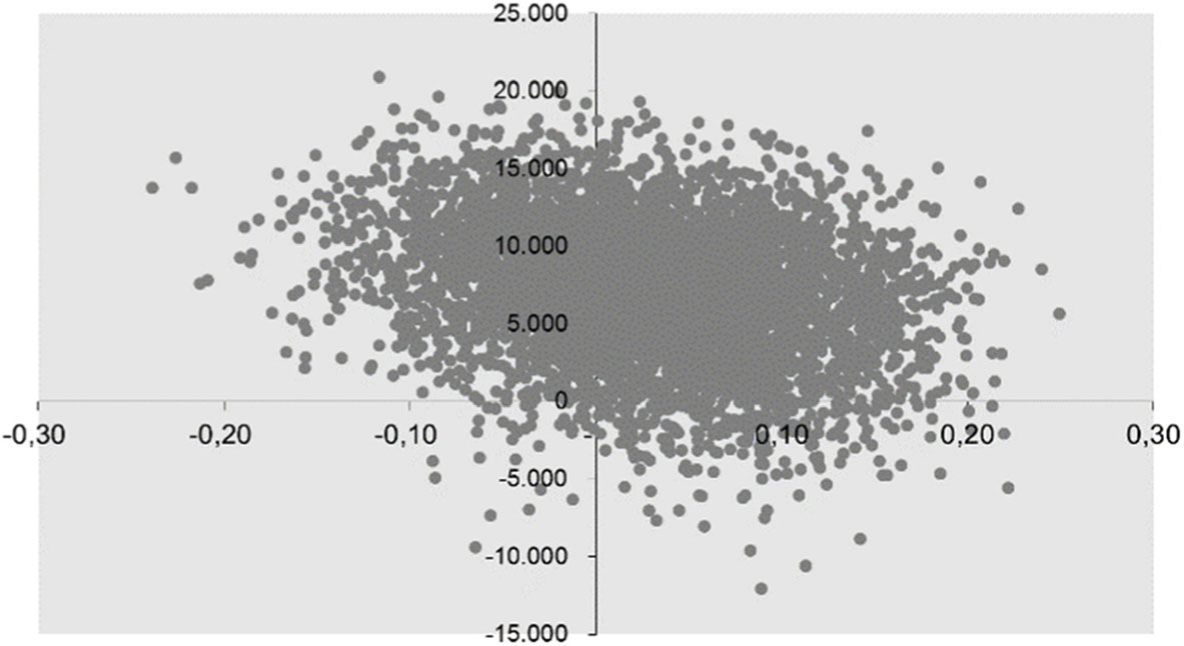
Cost-effectiveness plane step-down DBT versus outpatient DBT. The Y-axis represents additional effects. The X-axis represents additional costs

After 12 months, the average EQ-5D-3L score was .65 (SD = .33) in step-down DBT and .62 (SD = .28) in outpatient DBT. In step-down DBT, healthcare costs were higher: €19,899 (SD = 14,210) versus €12,472 (SD = 14,300). There were no differences in productivity costs, with €906 (SD = 3462) for step-down DBT and €964 (SD = 3633) for outpatient DBT. The ICER with imputed values was €278,067 per QALY. The acceptability curve showed that the intervention has a probability of 21% of being cost-effective if the maximum threshold is € 80,000. The ICER was recomputed to gauge the effect of the imputation process on the underlying data. The ICER was reduced to €220,566, which is still above the threshold. The bootstrap-data are shown in Fig. 3. The majority of the points (59%) lie in the north-east quadrant of the CE-plane. This indicates that step-down DBT is more effective in increasing quality of life, but also more costly, than outpatient DBT.

## Discussion

We conducted a pragmatic RCT to compare 9 months of step-down DBT to 12 months of outpatient DBT in a sample that reported severe levels of BPD. Step-down DBT consisted of 3 months of residential plus 6 months of outpatient DBT. Our main findings were that: a) the probability of suicidal behaviour did not change significantly over 12 months, b) the probability of NSSI decreased significantly in step-down DBT, but not in outpatient DBT, c) severity of borderline symptomatology decreased significantly in both groups, with the improvement leveling off at the end of treatment, and d) the extra costs per gained QALY in step-down DBT exceeded the €80,000 threshold that is considered acceptable for severely ill patients in the Netherlands.

In the step-down program, 40 out of 42 (95%) patients were willing to initiate DBT treatment. In the outpatient program, only 19 out of 42 (45%) patients were willing to initiate DBT treatment. The noncompliance in outpatient DBT may have introduced confounding. We reported that participants who were randomized to outpatient DBT had to wait longer before they met their therapist. It is possible that participants who were willing to wait, differed systematically from those who refrained from treatment or sought help elsewhere. Alternatively, it is plausible that step-down DBT reached a subsample that was unable to engage in an outpatient treatment [31]. However, we found no significant differences on key demographic or clinical variables between the starters in step-down DBT and outpatient DBT. On balance, the fact that initial randomization was undermined, poses a threat to the internal validity of our study. This means that the main findings should be considered tentative. In other words, the results of our study do not warrant shifting resources from step-down to outpatient DBT programs.

We also tested the hypothesis that a residential setting reinforces suicidal behaviour. This hypothesis was rejected. On the contrary, we found a significant decrease in the probability of suicidal behaviour during the first 3 months of step-down DBT (i.e. the residential phase). These findings are similar to those reported in studies of 3-month inpatient DBT [34, 47], and challenge the perspective that hospitalization always reinforces suicidal behaviour in people diagnosed with BPD. It seems that possible iatrogenic effects of hospitalization can be neutralized if the support staff is trained in DBT. Of note, only 35% of participants still engaged in NSSI after Bohus’ inpatient DBT-program [34]. In our residential DBT-program over 70% of the participants reported that they still engaged in NSSI during treatment. Percentages at baseline were almost identical: about 75% [34, 47]. This difference may be related to the timeframe that was used to measure NSSI. In Bohus et al., participants were asked to report NSSI that occurred in the last month. In our study, participants were asked to report NSSI that occurred in the last 3 months. Another plausible explanation is the difference in coaching after office hours. The hospital setting in Bohus et al. allowed for 24/7 crisis interventions by support staff. In our study, support staff were only present during office hours and on weekdays. Telephone consultation after office hours was within the limitations set by the therapist. Future research should take this into account, either by implementing this component of DBT as well, or by conducting a dismantling study first.

Some strengths of the present study are notable. First, we ensured that interventions were allocated by means of a concealed randomization procedure. Second, treatment adherence was evaluated in both conditions. Third, the protocol was published in advance and all analyses were performed by independent experts [32]. Fourth, our design has strong ecological validity given that it was performed in a nonacademic context. On the other hand, this project had several limitations. Foremost, the initial randomization was undermined by a high percentage of non-starters in outpatient DBT. Second, data collectors were not blind to the assigned intervention. Third, the skills training groups of outpatient DBT contained patients that did not participate in the study. Thus, the composition of the skills training groups in outpatient DBT differed from the groups in step-down DBT, which consisted only of study participants. Fourth, evaluation of treatment integrity showed that some sessions were non-adherent (DBT Expert Rating Scale scores < 4.0). A final limitation is the lack of follow-up data.

The effectiveness of step-down versus outpatient DBT for patients that report severe levels of BPD-symptoms remains to be established in future research. It will be equally important to assess which moderators (e.g. characteristics of the individual or his/her social context, treatment integrity, regional differences in mental health care organization and stigma) change the direction or strength of the relation between the treatment (step-down versus outpatient) and the outcome (e.g. NSSI, BPDSI, drop-out, QALY). Given the treatment outcomes we reported in this pragmatic RCT, treatment integrity, in particular, deserves further examination. Adherence to a protocol is essential for internal validity and generalizability of the results in our domain of research. However, it would be interesting to learn more about the relationship between treatment adherence and treatment outcome. Is it a linear relationship? Or does the added value lessen once a certain level of adherence is reached? Finally, yet importantly: the long-term effectiveness and cost-effectiveness of step-down DBT remain to be evaluated.

The main methodological challenges we encountered were noncompliance and attrition in outpatient DBT. We do not know whether these phenomena indicate that step-down DBT was more effective at engaging people suffering from severe levels of BPD [31]. To answer this question in future research, we need to rule out waiting list issues and strengthen participants’ commitment before randomization takes place. When these conditions are met, higher compliance in step-down DBT would provide support for Bloom’s hypothesis [31]. A second step would be to find out what predicts compliance in step-down and in outpatient DBT. We found no significant differences in demographic or clinical variables in our study. Perhaps factors that we did not include, such as social isolation, institutionalization, and marginalization, were paramount. A last consideration is that, even though interesting in its own right, noncompliance challenges the feasibility and validity of a RCT. We would suggest future researchers to consider a Zelen’s design, or to add a second control condition that would allow us to compare step-down DBT to ‘residential care as usual’ plus outpatient DBT [48].

## Conclusions

A pragmatic randomized controlled trial in the Netherlands showed that 9 months of step-down DBT is an effective treatment for people suffering from severe levels of BPD. However, step-down DBT was not more effective than 12 months of outpatient DBT, nor was it more cost-effective. These findings should be considered tentative because of relatively high noncompliance with the treatment assignment in outpatient DBT. Furthermore, the long-term effectiveness of step-down DBT, and moderators of treatment response, remain to be evaluated.

## Abbreviations

BPD: Borderline Personality Disorder
BPDSI: Borderline Personality Disorder Severity Index
DBT: Dialectical Behaviour Therapy
DSM: Diagnostic Statistical Manual of Mental Disorders
GLMM: Generalized Linear Mixed Model
ICER: Incremental Cost-Effectiveness Ratio
LMM: Linear Mixed Model
LPC: Lifetime Parasuicide Count
NSSI: Non-Suicidal Self-Injury
QALY: Quality Adjusted Life Year
RCT: Randomized Controlled Trial
SCID: Structured Clinical Interview for DSM Disorders
TIC-P: Treatment Inventory Cost in Psychiatric Patients
